# Electrolyte Disturbances Are Associated with Non-Survival in Dogs—A Multivariable Analysis

**DOI:** 10.3389/fvets.2017.00135

**Published:** 2017-08-18

**Authors:** Robert Goggs, Sage De Rosa, Daniel J. Fletcher

**Affiliations:** ^1^Department of Clinical Sciences, College of Veterinary Medicine, Cornell University, Ithaca, NY, United States; ^2^Department of Clinical Studies, University of Pennsylvania School of Veterinary Medicine, Philadelphia, PA, United States

**Keywords:** sodium, chloride, potassium, calcium, death

## Abstract

Electrolyte disorders have been individually associated with mortality in small populations of dogs and cats with specific conditions, but the associations and interactions between electrolyte disturbances and outcome have not been evaluated in a large, heterogeneous population. It was hypothesized that abnormalities of sodium, chloride, potassium, and calcium concentrations would be independently and proportionately associated with death from natural causes and with all-cause mortality in dogs. An electronic database containing 33,117 electrolyte profiles was constructed to retrospectively assess the association between disorders of sodium, potassium, corrected chloride, and ionized calcium concentrations with non-survival and with death excluding euthanasia by multivariable modeling. A second database containing 11,249 records was used to validate the models constructed from the first database. All four electrolytes assessed had non-linear U-shaped associations with case fatality rates, wherein concentrations clustered around the reference interval had the lowest case fatality rates, while progressively abnormal concentrations were associated with proportionately increased risk of non-survival (AUROC 0.624) or death (AUROC 0.678). Multivariable modeling suggested that these electrolyte disturbances were associated with non-survival and with death from natural causes independent of each other. This study suggests that measurement of electrolyte concentrations is an important component of the assessment of dogs in emergency rooms or intensive care units. Future studies should focus on confirming these associations in a prospective manner accounting for disease severity.

## Introduction

Several organ systems in dogs are dedicated to the physiological regulation of fluid status, electrolyte concentrations, and acid–base balance, including the kidneys, hypothalamus, and various parts of the cardiovascular system (1). Multiple homeostatic mechanisms manage perturbations in these parameters and return electrolyte concentrations to within physiological ranges (2). Despite these mechanisms, electrolyte disturbances are commonly encountered in veterinary emergency and critical care medicine (3–6). These disturbances are typically secondary to another condition that causes alterations in fluid balance or result from excessive gain or loss of particular electrolytes in excretions or effusions. Most electrolyte disorders are mild and are likely not life-threatening. Physiologically, marked disturbances in electrolyte concentrations have consequences for acid–base status (7, 8), regulation of membrane potentials (9), neuronal excitability (10), muscle contraction (11), and the function of enzyme systems (12), and hence are likely to be associated with an increased risk of death. Individually, disorders of sodium, potassium, chloride, and calcium have been associated with outcome in humans and in small animals.

Disturbances in plasma sodium concentrations are associated with death in critically ill people, independent of disease severity (13). Interestingly, even small changes in sodium concentration within the reference interval have been associated with increased mortality risk (14). In many cases, disorders of sodium are due to disorders of water balance (15), and hence fluid balance may in turn be partly responsible for the association between sodium disorders and outcome. It is now recognized in both human (16, 17) and veterinary medicine (18) that fluid overload is associated with adverse outcomes.

Regulation of chloride is crucial for the maintenance of osmolality and for acid–base balance (19). Decreases in plasma chloride increase strong ion difference (SID), causing a hypochloremic alkalosis, while increases in plasma chloride decrease SID, causing a hyperchloremic acidosis (19). Identification of such alterations can be clinically valuable to increase the index of suspicion for gastrointestinal obstruction (3) or the presence of unmeasured anions (20). In addition to concerns of the impact of chloride disorders on acid–base balance, recently there has also been considerable interest in the potential impact of iatrogenic hyperchloremia on outcome in critically ill people (21). While this is likely multifactorial, one putative contributor is the extensive use of 0.9% saline as a resuscitative fluid in human medicine (22, 23). It has been hypothesized that association between 0.9% saline administration and outcome relates to increased kidney injury in some patients (24).

Disorders of potassium are well known to be associated with a risk of death in veterinary patients. In particular, hyperkalemia is a common contributor to death in patients with kidney injury (25), urinary tract obstruction (26, 27), and hypoadrenocorticism (28, 29). Hypokalemia is also commonly encountered in veterinary medicine, although an association between hypokalemia and mortality has not been established outside of certain specific conditions (30, 31).

Both hypercalcemia and hypocalcemia are commonly encountered in emergency and critical care practice, with prevalence in some populations reportedly as high as 31% (32, 33). Hypercalcemia has been associated with mortality in critically ill small animals, likely due to its association with kidney injury (34), and cancer (35). Ionized hypocalcemia is frequently seen in dogs and cats with pancreatitis (36) and sepsis and is associated with increased mortality in these patients (37, 38).

In summary, individual electrolyte disorders have been studied in small populations of dogs and cats with specific conditions, but the associations between electrolyte disturbances and outcome have not been evaluated in a large, heterogeneous group of veterinary emergency and critical care patients. Furthermore, potential interactions between electrolyte disturbances and outcome have not been evaluated, such that we do not know if disturbances in individual electrolytes are independent predictors of mortality in the setting of disorders accompanied by more than one electrolyte abnormality. This study aimed to fill this knowledge gap by analyzing the association between electrolyte abnormalities and outcome in dogs using a multivariable analysis of a large database. It was hypothesized that abnormalities of sodium, chloride, potassium, and calcium concentrations would be independently and proportionately associated with death from natural causes and with all-cause mortality (including euthanasia) in dogs.

## Materials and Methods

### Electrolyte and Metabolite Analyses

Blood gas and electrolyte analyses were conducted using a point-of-care analyzer on heparinized blood samples (RapidPoint 405, Siemens, Malvern, PA, USA). Local reference intervals for the blood gas analyzer were previously generated from 20 normal dogs that were not part of the study population. These animals were considered healthy on the basis of history, physical examination, and the results of complete blood count and serum chemistry profiles. Serum chemistry analyses were conducted using an automated chemistry analyzer (Cobas ModP, Roche-Hitachi, Indianapolis, IN, USA). Sodium concentrations were not corrected for glucose concentration. Raw chloride values were available and chloride concentrations were also corrected for all profiles for changes in measured sodium concentration (39), using Eq. 1. The midpoint of the reference interval was used as the normal sodium concentration:

(1) $${\left[{\text{Cl}}^{-}\right]}_{\text{Corrected}}={\left[{\text{Na}}^{+}\right]}_{\text{Normal}}/{\left[{\text{Na}}^{+}\right]}_{\text{Measured}}\times \;{\left[{\text{Cl}}^{-}\right]}_{\text{Measured}}.$$

### Case Selection and Database Compilation

An electronic database of blood gas and electrolyte analyses conducted between 05/31/2007 and 01/03/2015 on patients presented to the institution emergency room or intensive care unit was searched for results from canine patients. The database was visually inspected and manually curated to remove samples from species other than dogs, samples with missing, erroneous or untraceable case numbers (e.g., 911), analyses from sample types other than blood (e.g., abdominal fluid), and analyses with missing data. Medical records from dogs with very high measured chloride concentrations were manually checked to identify patients receiving potassium bromide as an anticonvulsant. These cases were removed from the dataset. Provided the dataset was complete then multiple samples from a single animal were kept in the database. Institution computerized medical record systems were then searched for data on patient signalment, presenting complaint, final diagnosis, outcome, and hospitalization dates. Four separate databases were thereby created containing the electrolyte data, point-of-care analyses, biochemistry analyses, and case demographics. A custom application (Visual Basic, Microsoft Visual Studio for Windows, Microsoft, Redmond, WA, USA) was written to search each database *via* the unique patient identifier in order to create a final composite database containing the relevant data from each of the separate databases. The entries in the final database corresponded to the time and date stamp from the blood gas and electrolyte analyses. The final database was then manually checked for accuracy by cross-referencing the database entries with the parent data sources for a randomized selection of cases, spanning the entire range of case numbers, and representing 0.1% of the total case entries.

### Test Database Compilation

A second electronic database of blood gas and electrolyte analyses conducted between 01/04/2015 and 01/03/2017 on patients presented to the institution emergency room or intensive care unit was generated in an identical manner to that above. This second database was used to assess the predictive ability of the multivariable model developed using the larger initial database. This was undertaken by calculating the predicted mortality probability for each profile in the second (test) database using the multivariable model generated from the first database, and through construction of a receiver operating characteristic (ROC) curve to evaluate model discrimination.

### Statistical Analyses

Prior to test selection, variables were tested for normality using the D’Agostino Pearson test, and appropriate descriptive statistics calculated. Parametric variables are presented as mean ± SD, while non-parametric variables are presented as median (min–max). Non-parametric continuous variables were compared using the Mann–Whitney *U* test and with box and whisker plots. To evaluate mortality in dogs with deviations of electrolyte concentrations from normal, electrolyte concentrations were banded into bins spanning the whole population. For sodium and chloride concentrations, these bins were 2 mmol/L wide, while for potassium concentrations, the bins were 0.28 mmol/L wide and for calcium they were 0.05 mmol/L wide. The case fatality rates for each of these bands of electrolyte concentrations were calculated and the percent case fatality plotted against the electrolyte concentration. Alpha was set at 0.05. Statistical analyses were conducted using commercial software (Prism 7 for Mac OS X, GraphPad Software, La Jolla, CA, USA; SPSS Statistics 24, IBM, Armonk, NY, USA).

## Results

### Demographics

The electrolyte data for the whole population are summarized in Table 1. The population consisted of 41.1% castrated males (*n* = 13,611), 38.4% spayed females (*n* = 12,716), 11.3% intact males (*n* = 3,742), and 9.2% intact females (*n* = 3,047). Overall, the case fatality rate in the whole population was 18.52%, while the death rate (excluding euthanasia) was 3.3%. Breed was recorded for 17,802 profiles (53.8% of the database). Mixed breed dogs were most common (*n* = 3,863, 21.7%). There were 149 pure breeds of dog recorded. The top 15 pure breed dogs were Labrador retriever (*n* = 1,778, 10.0%), golden retriever (*n* = 715, 4.0%), German shepherd (*n* = 590, 3.3%), dachshund (*n* = 489, 2.5%), Yorkshire terrier (*n* = 428, 2.4%), shih tzu (*n* = 395, 2.2%), rottweiler (*n* = 355, 2.0%), beagle hound (*n* = 343, 1.9%), poodle (*n* = 336, 1.9%), boxer (*n* = 313, 1.8%), miniature schnauzer (*n* = 291, 1/6%), English bulldog (*n* = 274, 1.5%), cocker spaniel (*n* = 261, 1.5%), pug (*n* = 255, 1.4%), and Maltese (*n* = 244, 1.4%).

**Table 1:** Summary electrolyte data.

Parameter	All dogs (*n* = 33,117)	Survivors (*n* = 26,984)	Non-survivors (*n* = 6,133)	Died (*n* = 920)
Age (years)	7 (4–10) [0–24]	7 (3–10) [0–24]	8 (5–11) [0–21]	8 (5–10) [0–17]
Na^+^ (mmol/L)	147.3 (144.3–150.3) [100.8–198.5]	147.3 (144.5–150.1) [100.8–190.5]	147.3 (143.5–151.2) [109.5–198.5]	147.5 (143.9–151.2) [111.5–187.8]
K^+^ (mmol/L)	4.08 (3.76–4.43) [0.97–14.80]	4.08 (3.78–4.41) [1.42–14.80]	4.08 (3.68–4.51) [0.97–11.34]	4.08 (3.78–4.41) [2.12–10.81]
Cl^−^ (mmol/L)	113.0 (110.0–117.0) [65.0–140.0]	113.0 (110.0–117.0) [65.0–140.0]	114.0 (109.0–119.0) [71.0–140.0]	116.0 (111.0–121.0) [79.0–140.0]
Corrected Cl^−^ (mmol/L)	114.0 (110.9–117.5) [72.1–143.8]	113.8 (110.8–117.2) [72.1–143.8]	114.8 (111.1–118.9) [81.0–142.0]	116.2 (112.1–120.4) [81.0–130.5]
iCa^2+^ (mmol/L)	1.27 (1.21–1.32) [0.20–2.53]	1.27 (1.21–1.32) [0.20–2.53]	1.24 (1.17–1.30) [0.33–2.51]	1.21 (1.14–1.28) [0.33–2.36]

*Descriptive statistics for age and the electrolytes for all dogs, survivors only, non-survivors (euthanized and died), and dogs that died only. All parameters were non-parametric and thus are summarized as median (interquartile range) [minimum–maximum]*.

### Individual Electrolytes

All four of the electrolytes assessed displayed similar non-linear U-shaped relationships with outcome. Broadly speaking electrolyte values close to the median were associated with the lowest case fatality rates, while deviations from the median were associated with increases in fatality rates proportionate to the deviation. These relationships were visible in the whole dataset and after dogs that were euthanized were excluded (Tables S4 and S5 in Supplementary Material).

**Figure 1:**
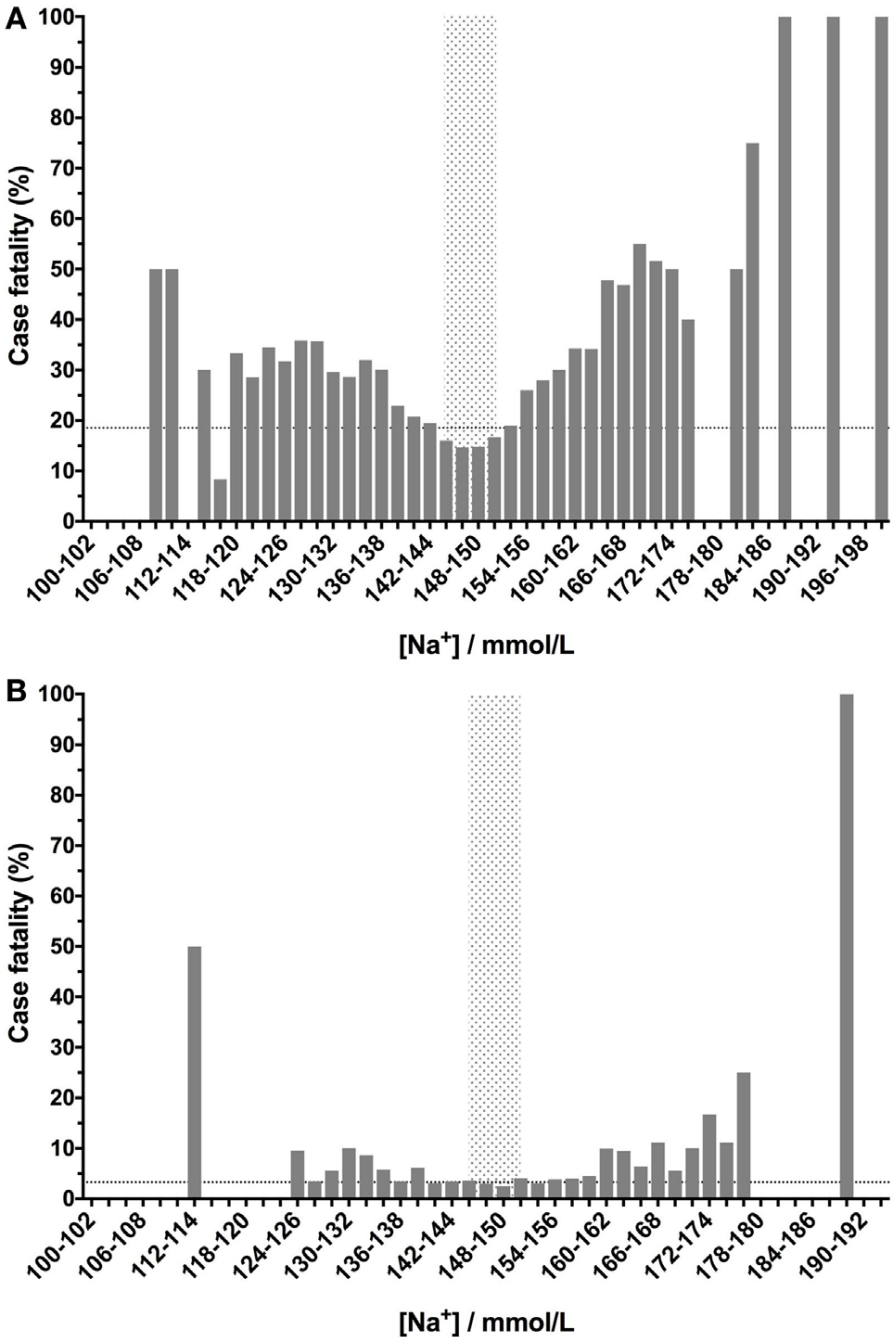
Canine blood sodium concentrations have a U-shaped relationship with case fatality rates. Low- or high-sodium concentrations are associated with increased case fatality rates compared to values within the reference interval (gray-shaded box). Sodium concentrations were banded into 2 mmol/L bins, and the percentage case fatality for the patients in each of these subgroups was calculated. The dotted lines denote the case fatality rate across the entire data set (the background case fatality rate). Panel **(A)** represents data from all samples (*n* = 33,117), including those from patients that were euthanized, while panel **(B)** represents data from patients that survived or died only (i.e., euthanized patients were excluded) (*n* = 27,904).

For sodium, dogs with concentrations within the reference interval (145–151 mmol/L) had case fatality rates below the background mortality rate for the whole population, but all dogs with values above or below the reference interval had a mortality rate above that of the whole population (Figure 1A). Once euthanized dogs were excluded, the protective effect of a normal value became more ambiguous, as did the overall U-shaped relationship. Despite the reduction in patient numbers in each bin, once sodium values increased or decreased by more than 5 mmol/L from the boundaries of the reference interval, the case fatality rates were consistently above the case fatality (3.3%) for the population (Figure 1B).

**Figure 2:**
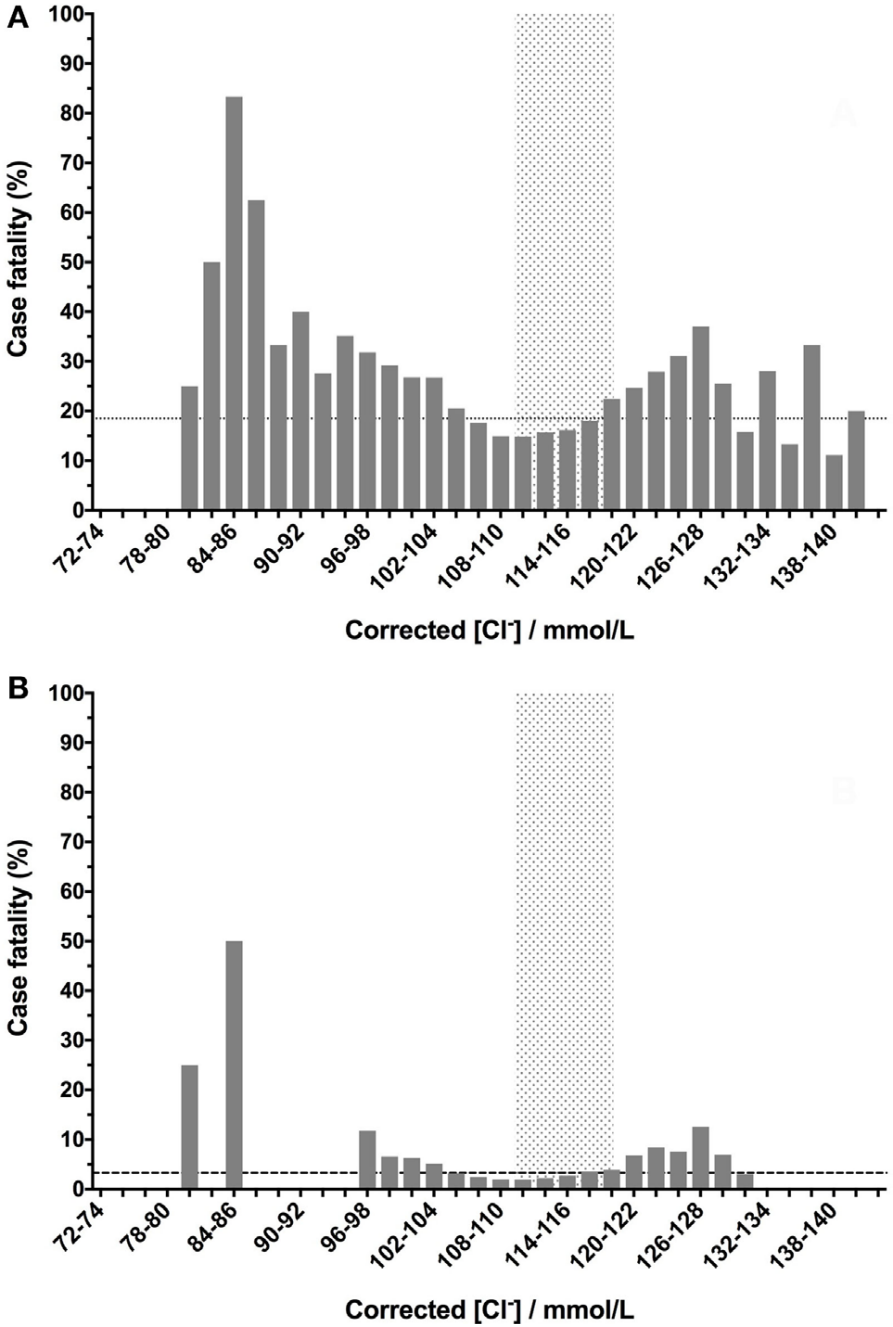
Canine corrected blood chloride concentrations have a U-shaped relationship with case fatality rates. Chloride values were corrected for sodium concentrations as follows: [Cl^−^]_Corrected_ = [Na^+^]_Normal_/[Na^+^]_Measured_ × [Cl^−^]_Measured_. Low- or high-corrected chloride concentrations are associated with increased case fatality rates. Corrected chloride values were banded into 2 mmol/L bins, and the percentage case fatality for the patients in each of these subgroups was calculated. The dotted lines denote the case fatality rate across the entire data set (the background case fatality rate). Panel **(A)** represents data from all samples (*n* = 33,117), including those from patients that were euthanized, while panel **(B)** represents data from patients that survived or died only (i.e., euthanized patients were excluded) (*n* = 27,904).

Corrected chloride values had a prominent U-shaped relationship with case fatality that was apparent in the whole population and in only those dogs that died or survived. In comparison to sodium concentrations, dogs with corrected chloride values within the reference interval and up to 4 mmol/L below the lower bound of the interval had case fatality rates below that of the whole population (Figure 2A). The number of dogs in the categories with markedly abnormal chloride concentrations was limited, but the data suggest that low corrected chloride values may have higher fatality rates than equivalent magnitudes of deviation from normal above the reference interval. These two effects were also present in the smaller population of dogs after euthanized dogs were excluded (Figure 2B).

**Figure 3:**
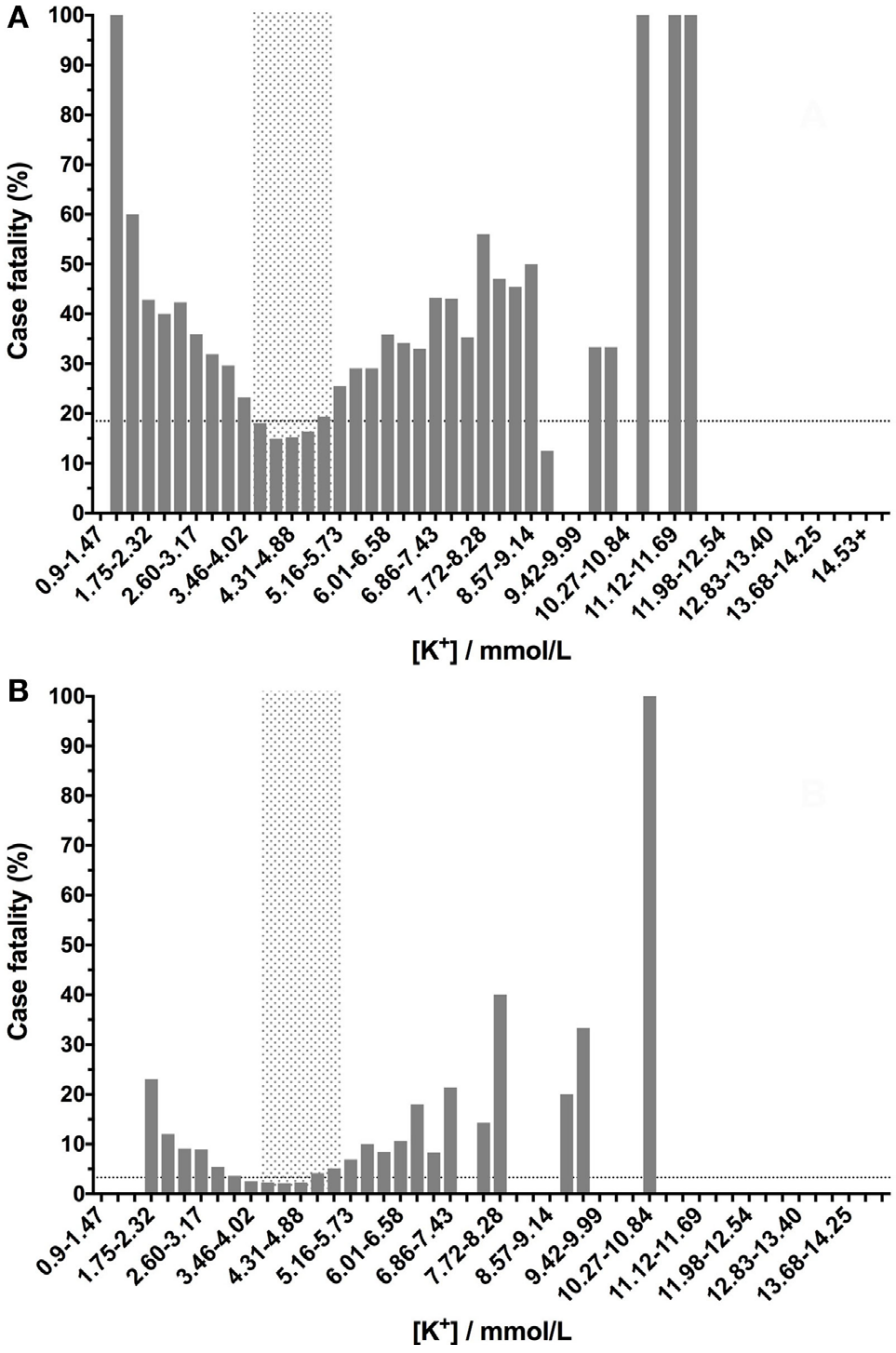
Canine blood potassium concentrations have a U-shaped relationship with case fatality rates. Low- or high-potassium concentrations are associated with increased case fatality rates. Blood potassium values were banded into 0.57 mmol/L bins, and the percentage case fatality for the patients in each of these subgroups was calculated. The dotted lines denote the case fatality rate across the entire data set (the background case fatality rate). Panel **(A)** represents data from all samples (*n* = 33,117), including those from patients that were euthanized, while panel **(B)** represents data from patients that survived or died only (i.e., euthanized patients were excluded) (*n* = 27,904).

As would be expected from a physiological perspective, the relationship between potassium concentration and case fatality rates was present over a much narrower range of concentrations than for sodium or chloride. As was the case for sodium, dogs with potassium concentrations in the reference interval had lower case fatality rates in both the whole population (Figure 3A) and after exclusion of euthanized dogs (Figure 3B). In contrast to sodium and chloride, dogs with potassium concentrations at either extreme had very high case fatality rates.

**Figure 4:**
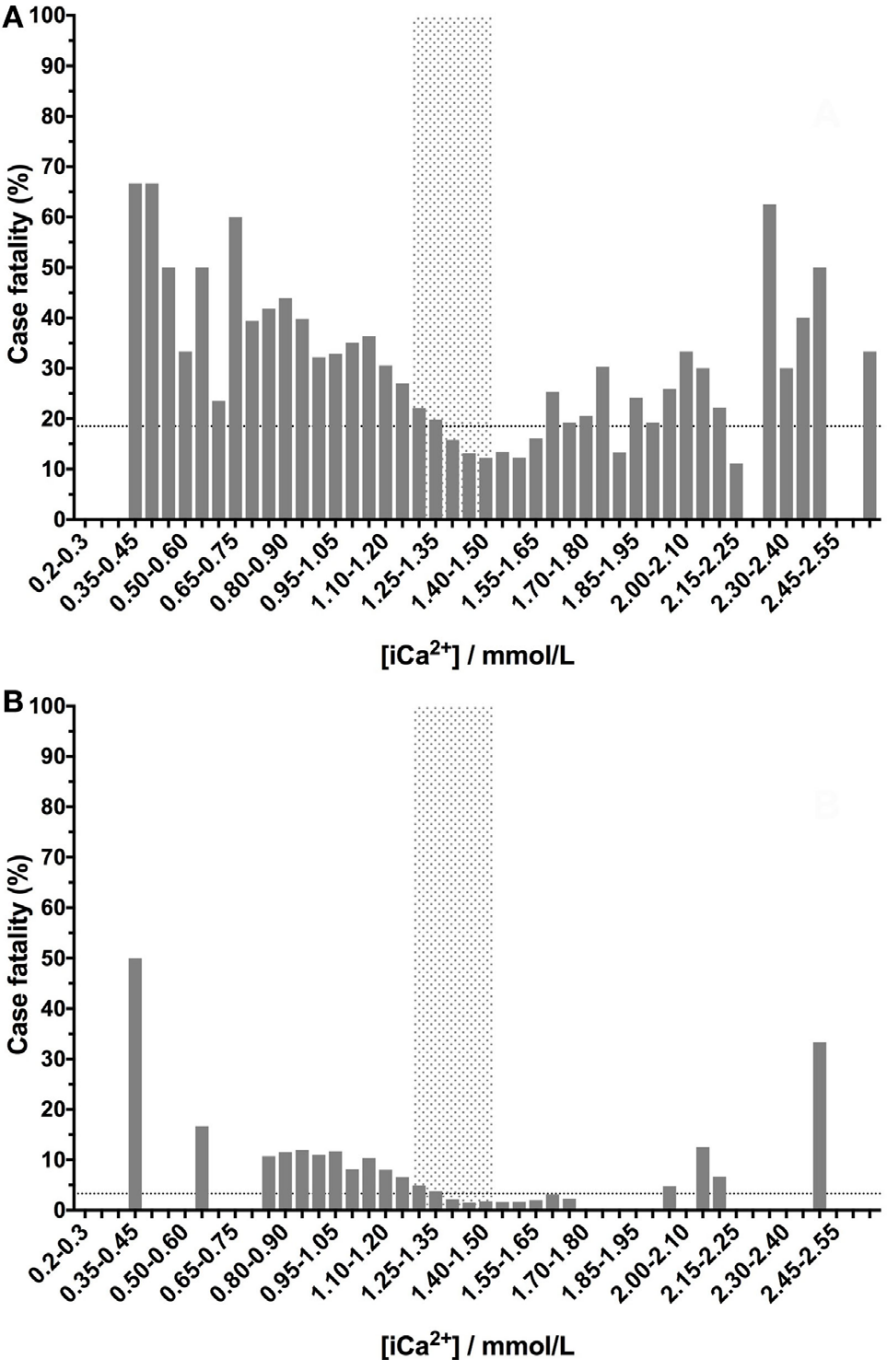
Canine ionized calcium concentrations have a U-shaped relationship with case fatality rates. Low- or high-ionized calcium concentrations are associated with increased case fatality rates. Ionized calcium values were banded into 0.05 mmol/L bins, and the percentage case fatality for the patients in each of these subgroups was calculated. The dotted lines denote the case fatality rate across the entire data set (the background case fatality rate). Panel **(A)** represents data from all samples (*n* = 33,117), including those from patients that were euthanized, while panel **(B)** represents data from patients that survived or died only (i.e., euthanized patients were excluded) (*n* = 27,904).

The relationship between ionized calcium and case fatality was U-shaped as for the other electrolytes, but there was a more prominent increase in mortality in dogs with low concentrations compared to high concentrations. In contrast to the other electrolytes, some dogs with calcium concentrations in the reference interval had higher case fatality rates compared with the background fatality rate of the whole population. This was the case for the whole dataset (Figure 4A) and after euthanized dogs were excluded (Figure 4B).

**Figure 5:**
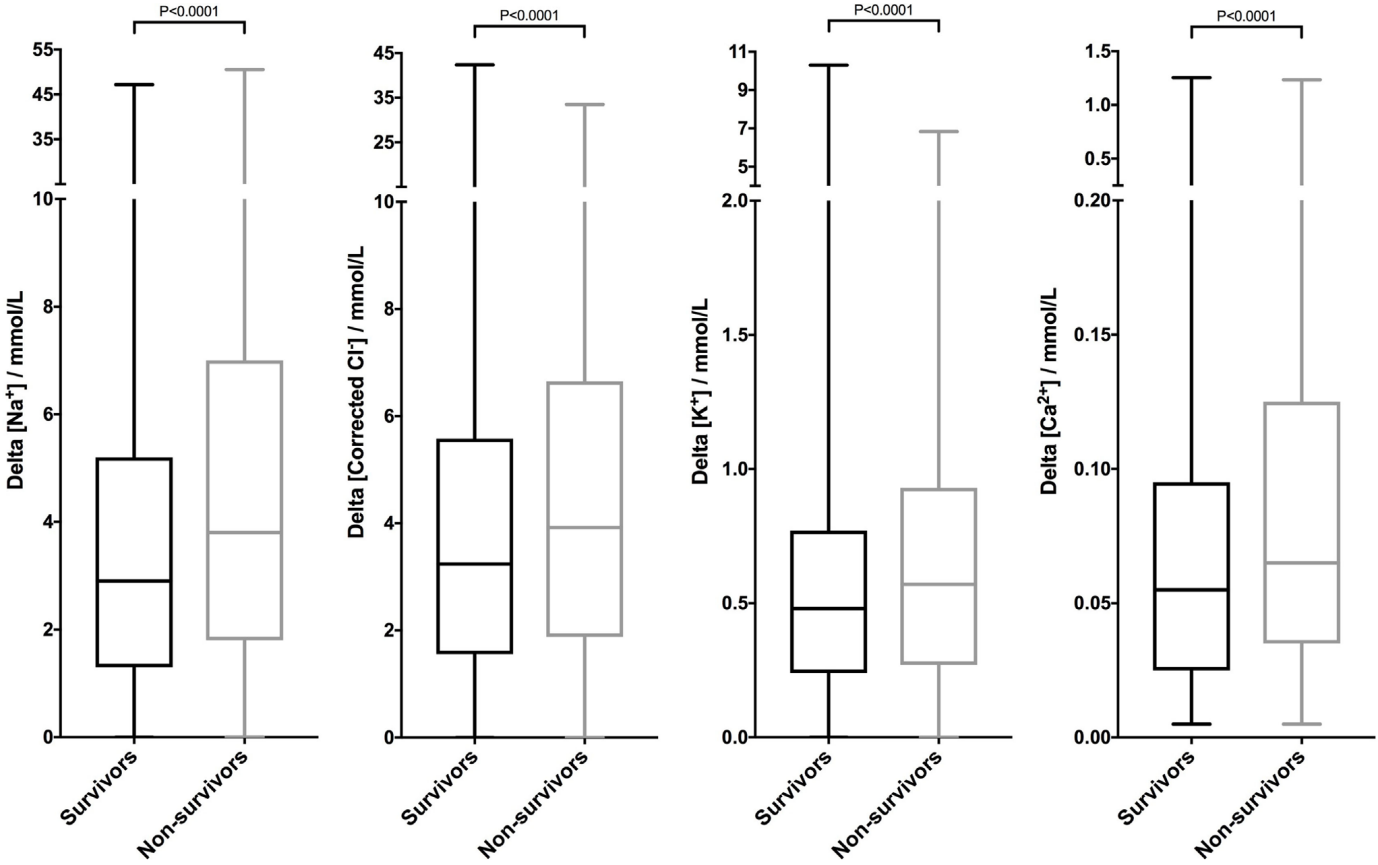
Canine electrolyte values are significantly different between survivors and non-survivors. Box and whisker plots of the deviations between patient sodium, corrected chloride, potassium, and ionized calcium values from the midpoints of their reference intervals were constructed from a database of 33,117 canine samples. The delta electrolyte concentrations were plotted according to outcome. Patients that died or were euthanized were classified as non-survivors. Patients that were discharged alive were classified as survivors. The delta electrolyte values for all four electrolytes were significantly larger for non-survivors compared to survivors (all *P* < 0.0001 by Mann–Whitney *U* test).

Due to the non-linear relationships between electrolyte concentrations and case fatality rates, comparing the median values of survivors and non-survivors did not produce meaningful analyses. However, calculation of the delta value (absolute value of the difference between measured value and reference interval midpoint) made it possible to compare the divergence from normal electrolyte concentrations in survivors and non-survivors. The delta electrolyte values were non-parametric (Table 2). Non-survivors (including dogs euthanized) had significantly larger delta electrolyte values compared to survivors. This was true for all four of the electrolytes assessed (*P* < 0.0001 by Mann–Whitney *U* test) (Figure 5).

**Table 2:** Summary delta electrolyte data.

Parameter	Survivors (*n* = 26,984)	Non-survivors (*n* = 6,133)	Died (*n* = 920)
Delta_Na^+^ (mmol/L)	2.9 (1.3–5.2) [0.0–47.2]	3.8 (1.8–7.0) [0.0–50.5]	3.7 (1.7–6.5) [0.0–39.8]
Delta_K^+^ (mmol/L)	0.48 (0.24–0.77) [0.00–10.30]	0.57 (0.27–0.93) [0.00–6.84]	0.69 (0.35–1.06) [0.00–6.31]
Delta_Corrected Cl^−^ (mmol/L)	3.24 (1.56–5.58) [0.00–42.38]	3.92 (1.88–6.65) [0.00–33.47]	4.40 (2.02–7.34) [0.01–33.47]
Delta_iCa^2+^ (mmol/L)	0.055 (0.025–0.095) [0.005–1.255]	0.065 (0.035–0.125) [0.005–1.235]	0.075 (0.035–0.145) [0.010–1.090]

*Descriptive statistics for the delta electrolyte parameters that represent the absolute difference (increase or decrease) between the measured electrolyte concentrations and the midpoint of the respective reference intervals for all survivors, non-survivors (euthanized and died), and dogs that died only. All parameters were non-parametric and thus are summarized as median (interquartile range) [minimum–maximum]*.

### Multivariable Logistic Regression

**Figure 6:**
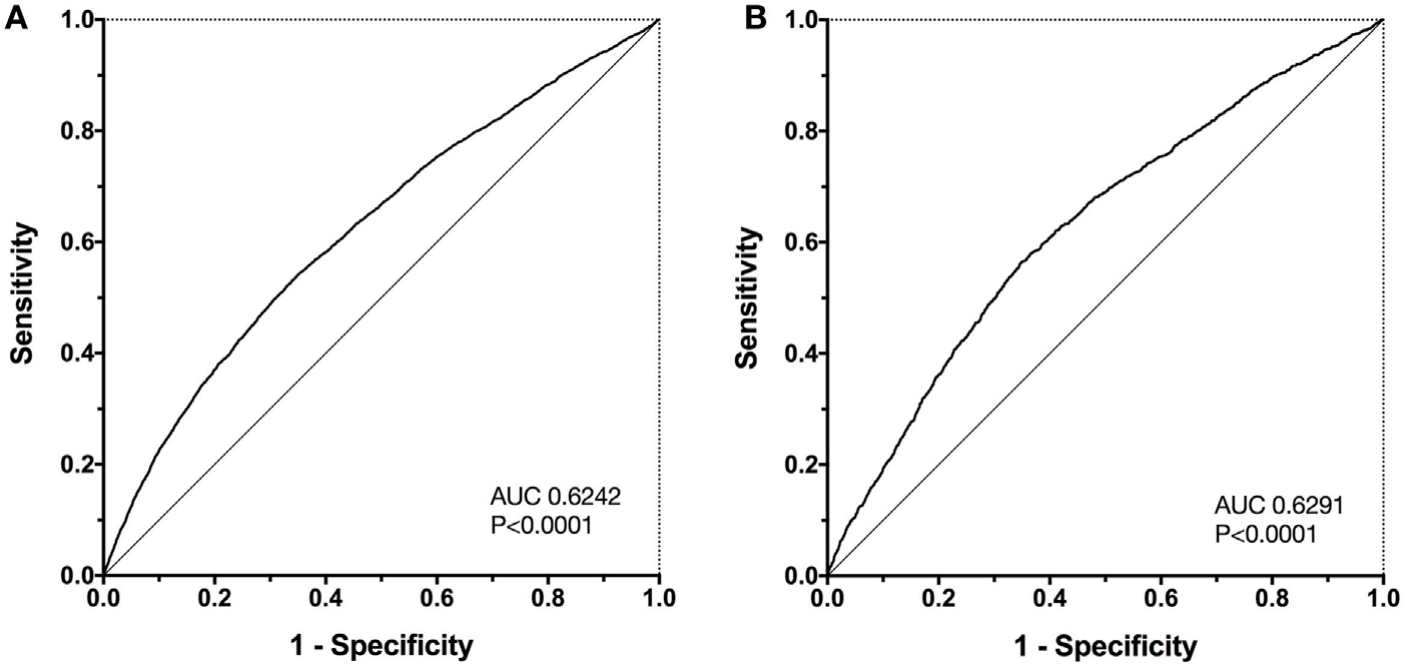
Receiver operating characteristic (ROC) curves for the survival probabilities as calculated by the final four-parameter multivariable model. Panel **(A)** displays the ROC curve for the initial population (*n* = 33,117). The area under the ROC curve (AUC) is 0.6242, significantly different from 0.5 (*P* < 0.0001). The same multivariable model was then used to predict outcomes in a separate test population of dogs from the same institution (*n* = 11,249), panel **(B)**. In the test population, the AUC was almost identical to that in the original population AUC 0.6291, significantly different from 0.5 (*P* < 0.0001).

All four of the delta electrolyte variables were associated with outcome. None of these variables exhibited collinearity and hence were identified as possible candidate predictor variables from the univariate analysis. These four variables were entered into a survival multivariable analysis in a forward stepwise fashion and retained in the final model if significantly (*P* < 0.05) associated with outcome. The final model contained all four delta electrolyte variables, as independent predictors of survival (Table 3). The equation of the model was ln(odds ratio) = 0.053(Delta_Na^+^) + 0.039(Delta_Cl^−^) + 0.318(Delta_K^+^) + 1.422(Delta_Ca^2+^) − 2.207, where all delta electrolytes are measured in mmol/L. The Hosmer–Lemeshow chi-square value for this model was 55.7, which was significant *P* < 0.001, indicating that the model was not well fitted. The Nagelkerke *R*^2^ value was 0.043, indicating that the model explained only 4.3% of the variability in the data. However, construction of a ROC curve for the outcome probabilities predicted by the model suggested the model was predictive of outcome (AUROC 0.6242, *P* < 0.0001) (Figure 6).

**Table 3:** Final multivariable model.

Variable	Coefficient	SE	Wald chi^2^	df	*P*-value	Odds ratio	95% CI lower	95% CI upper
Delta_Na^+^	0.053	0.003	273.378	1	<0.001	1.055	1.048	1.061
Delta_Cl^−^	0.039	0.004	101.255	1	<0.001	1.040	1.032	1.048
Delta_K^+^	0.318	0.030	114.192	1	<0.001	1.375	1.297	1.458
Delta_iCa^2+^	1.422	0.127	124.491	1	<0.001	4.144	3.228	5.319
Constant	−2.207	0.030	5,247.145	1	<0.001	0.110		

*The output from the multivariable modeling to predict outcome using deviations of electrolyte concentrations from the midpoints of their respective reference intervals as predictor variables. These predictor variables are labeled Delta_electrolyte to indicate that they represent deviations from normal in either direction (hyper- or hypo-)*.

*df, degrees of freedom of the chi^2^ test; CI, confidence interval*.

**Figure 7:**
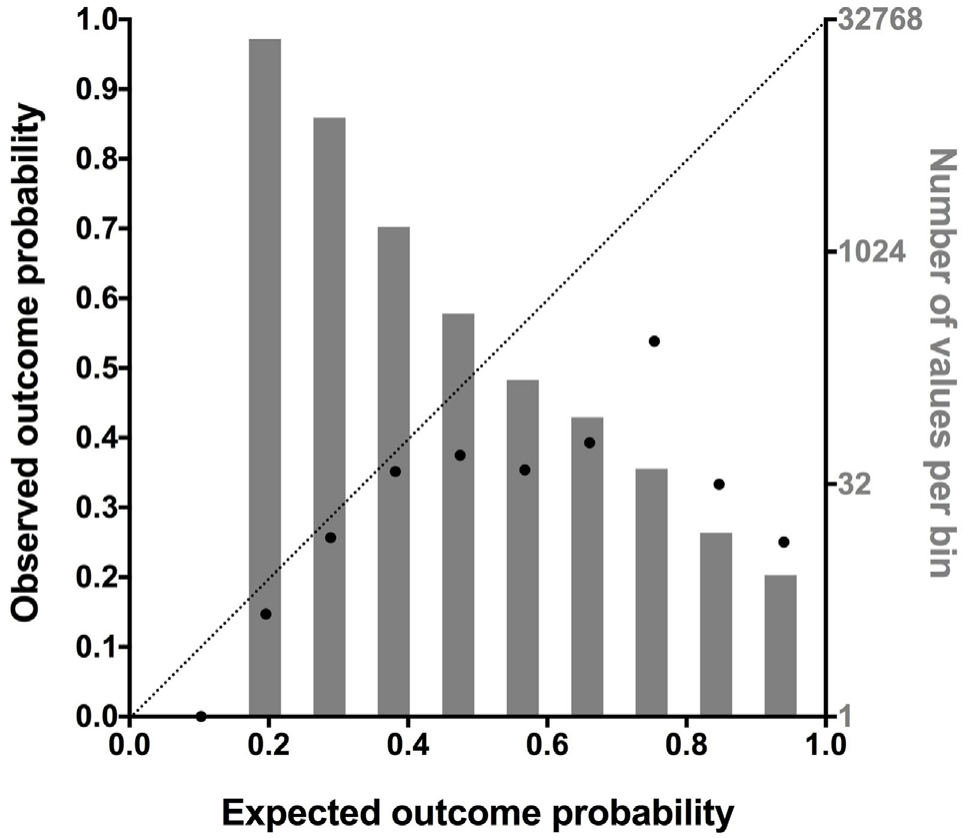
A combination plot comparing the predicted case fatality probability divided into deciles against the observed case fatality probability (black dots, abscissa, and left ordinate). This analysis was conducted to further assess the cause of the poor model fit. A value of 0.0 indicates 0% fatality, a value of 0.5 indicates 50% fatality, and a value of 1.0 indicates 100% fatality. A line of identity is also plotted to aid interpretation. As can be seen from comparing the line of identity to the black dots, as the mortality rate predicted by the model increases above 0.5, the correlation between predicted and observed mortality decreases. This suggests the model overestimated the likelihood of fatality as the probability increases. As indicated by the number of profiles in each of the deciles (gray bars, right ordinate, log-2 scale), there were many cases with low predicted mortality but few cases with a high predicted mortality.

Further analysis of the model calibration was performed by plotting the predicted case fatality probability divided into deciles against the observed case fatality probability (Figure 7). This suggested that the explanation for the poor model fit was an overestimate of case fatality probability as the probability increases, which corresponded to low numbers of cases in the upper probability deciles. This suggests that the model may overestimate the likelihood of fatality in part due to the low numbers of dogs with high-predicted fatality rate.

The predictive ability of the multivariable model was then tested by calculating the predicted mortality probability for each profile in the second (test) database containing 11,249 profiles. A ROC curve was then constructed to assess the discriminating ability of the model in this population. In the test population, where the overall case fatality rate was 15.29%, the AUROC was 0.6291, *P* < 0.0001.

To confirm that the association between outcome and electrolyte disturbances was not biased by including dogs that were euthanized, the multivariable analysis was repeated using a dataset including only survivors and dogs that died (*n* = 27,904). The final model of predicting death included all four electrolytes as independent predictors. The equation of this model was ln(odds ratio) = 0.028(Delta_Na^+^) + 0.051(Delta_Cl^−^) + 0.551(Delta_K^+^) + 1.662(Delta_Ca^2+^) − 4.228, where all delta electrolytes are measured in mmol/L. The Hosmer–Lemeshow chi-square value for this model was 44.4, which was significant *P* < 0.001, indicating that the model was not well fitted. The Nagelkerke *R*^2^ value was 0.038. Construction of a ROC curve for the outcome probabilities predicted by the model suggested the model was more discriminating for outcome when considering only animals that died (excluding animals that were euthanized) (AUROC 0.678, *P* < 0.0001) (data not shown).

## Discussion

Disturbances in individual electrolytes in specific patient populations have been previously associated with mortality in various veterinary and human studies (40). Most of those studies have associated specific disease processes or situations in which increases or decreases in an electrolyte was associated with outcome (41). This study confirms these previous findings and adds to this body of literature by identifying associations between electrolyte disturbances and outcome in two very large and heterogeneous groups of dogs. Furthermore, this study demonstrates that both decreases and increases in electrolyte concentrations proportionately increase the risk of non-survival (including euthanasia) and of death (excluding euthanasia). This study also suggests that distinct and separate electrolyte disturbances have a cumulative effect on mortality risk. In other words, patients with more than one disturbance in either direction are at greater risk of a poor outcome irrespective of the combination of electrolyte disturbances present, consistent with a recent study of human ICU patients (42).

Multivariable logistic regression models enable statistical assessment of the relationship between explanatory variables (deviation in electrolyte concentrations from normal) and the outcome variable (all-cause mortality or death from natural causes). The equation of the resulting model provides an estimate of the likelihood of the outcome as a natural log of the odds ratio. The equation incorporates a series of coefficients or weightings of the impact of changes in the predictors. To use the equation for any given patient, the deviation in electrolyte concentrations are calculated, the figures are multiplied by the relevant coefficients. The probability of the outcome is then calculated by raising *e* to the power of the value calculated by the equation.

Given the retrospective nature of this study, it is only possible to speculate about the causes of the associations between electrolyte disturbances and outcome; however, irrespective of cause, marked variations in electrolyte concentrations will negatively impact cellular processes that underpin tissue and organ function. For instance, hyperkalemia alters the resting membrane potential of excitable cells, which can manifest as bradycardia due to atrial standstill and slowed conduction. Ionized hypocalcemia may cause a decrease in the availability of calcium in the extracellular fluid, reducing the contractile abilities of skeletal and cardiac muscle tissue and causing vasoplegia. Marked changes in sodium concentration affect volume regulation at the level of individual cells and within the body as a whole. Marked alterations in chloride concentration cause acid–base disturbances with secondary consequences for enzymatic processes and ion channel regulation. At the extremes, these electrolyte changes may be the primary cause of death in some patients. However, the data suggest that even more modest alterations in ion concentrations can affect outcome, possibly due to additive effects of dysregulation of ion concentrations on normal cellular functions. Our study was also limited by the nature of the output from our medical records system, which precluded easy analysis of the recorded diagnoses. Many patients also had multiple diagnoses, which would likely obscure the origin of any electrolyte disturbances present.

The results of this study have implications for veterinary clinical practice. Since the study design precludes identification of a causal association between electrolyte disturbances and outcome, the data presented here should not be used to guide specific treatment decisions to correct electrolyte disturbances. However, the strength of the association between death and electrolyte abnormalities should heighten the concern that clinicians have for patients with such disturbances. Taken in context of other literature, the data presented here suggest that clinicians should take steps to identify and manage the underlying disease causing the electrolyte abnormality. Arbitrarily seeking to normalize the electrolyte concentrations in individual patients cannot be recommended, however, since they may simply be markers of another process that requires treatment primarily. Clinicians should be wary of attempting to achieve “euboxia” (normalization of all boxes in a clinical pathology printout) with unintentional consequent detrimental effects (43–45). Although the data suggest that the magnitude of risk increases in a non-linear fashion as the level of the disturbance becomes more extreme, it is also clear that small disturbances in electrolyte concentrations even within the reference interval may have consequence. This is consistent with similar electrolyte data from people (40) and is akin to recent increased awareness that subclinical acute kidney injury (AKI) can be identified by change in creatinine concentration that remain within the reference interval (46, 47). This step-change in mindset has encouraged clinicians to take a more proactive approach to the management of patients with subclinical AKI (48). This study suggests the same perspective should be adopted when subtle electrolyte changes are encountered.

As previously discussed, this study was retrospective and hence cannot establish causation—only association. Retrospective studies such as this can be confounded by unknown and unmeasured variables, temporal relationships such as the impact of therapy or stage of disease, or by biases introduced by selection or misclassification. The study populations were very heterogeneous, including any patient that underwent blood gas and electrolyte analysis in the emergency room or intensive care unit at our institution. This is likely to have increased the number of dogs with mild illness severity and will have included a diverse group of patients with multiple other risk factors for mortality. Although it was possible to demonstrate that the electrolyte disturbances were independent of each other, it was not possible to determine if the electrolyte disturbances identified were independent of illness severity. In similar studies of human ICU patients, the near universal availability of validated illness severity scores such as APACHE II or the SOFA score enable the level of injury or disease to be accounted for in multivariable analyses (14, 21). It was not possible to calculate any of the established veterinary illness severity scores such as SPI2 or the canine APPLE score in this study due to a lack of availability of the necessary data within our electronic medical records. Thus, it is possible that the associations observed in this study represent epiphenomena associated with illness severity.

Future studies gathering data prospectively might be able to overcome this issue and confirm that electrolyte disturbances are independently associated with outcome. At this time, we can only speculate that this is the case. The very large datasets that we employed may have helped to reduce the impact of the case heterogeneity in the population. Using a dataset of more than 33,000 records enabled identification of even small signals. The Nagelkerke’s *R*^2^ value from the multivariable model was very low, suggesting that less than 5% of the variability in the data were explained by the four predictor variables included. This is still noteworthy, however, since it implies that even with all of the other potential explanations for the cause of death or euthanasia in the population electrolyte disturbances alone account for 5% of the risk of non-survival.

The datasets evaluated in this study included more than one profile from some patients. This has the potential to bias the results, by increasing the strength of the outcome signal (toward survival or non-survival) from individual patients. This bias could make it either more or less likely that an association between electrolyte concentration and poor outcome was identified. It is possible that multiple samples might be taken from a patient with normal electrolytes that did not survive, or from a patient with abnormal electrolytes that did survive. It is possible that more samples may have been collected from sicker patients that may have been more likely to have electrolyte disturbances. It was not possible to determine the impact that this may have had on the analyses. One strategy would be to eliminate any instances of multiple samples from the same patient. However, this would likely have two effects, to reduce the size of the database and to introduce another type of bias by forcing selection of one of the profiles for inclusion over the others. The potential impact of this selection bias is hard to predict, and hence it was not performed.

In addition to including multiple profiles from a single patient, it was also not possible to eliminate the impact of therapy on the measured electrolyte concentrations or on outcome. Thus, patients may have received therapy including fluid administration before or after the electrolyte profile was recorded. This is important because administration of high-chloride concentration fluids has been associated with an increased risk of death in people (49, 50). Each outcome event recorded in the database was linked to the electrolyte profiles from that hospital visit, however, so if therapy had a detrimental or a positive effect on outcome, then these effects would need to have lasted the entire hospital visit in order to affect the association between the recorded electrolyte profile and the outcome. Typically, patients with abnormal profiles would have a repeat sample analyzed after therapy. This too may have blunted the effect of therapy on the associations we identified because abnormal profiles that normalized with therapy such that the patient survived would not be associated with non-survival. This implies that despite therapy, disturbances in electrolyte concentrations are proportionately associated with the risk of death.

The database did include many more patients with a low risk of death compared to a high risk of death, and likely reflects the overall population of cases seen at our institution. This may have detrimentally affected the multivariable modeling, as could be seen from the Hosmer–Lemeshow calibration test (51). The low numbers of cases with very marked electrolyte disturbances and the low numbers of very sick patients likely affected the accuracy of the estimates of mortality risk at the extremes. The impact of euthanasia is difficult to address in all veterinary studies incorporating mortality as an outcome measure. Some of the analyses reported in this study incorporated all patients, while others specifically excluded patients that were euthanized. Importantly, the principal non-linear U-shaped relationships between case fatality rates and electrolyte disturbances remained even after exclusion of dogs that were euthanized. Admittedly, the numbers of cases in these analyses were reduced compared to the whole dataset, but they suggest that electrolyte disturbances are associated with mortality from natural causes. In addition, the multivariable analysis performed on the dataset after exclusion of euthanized dogs was more actually discriminant, based on AUROC than in the larger population.

In summary, this study suggests that disturbances in sodium, potassium, corrected chloride, or ionized calcium concentrations above or below the midpoint of the reference interval are associated with death in dogs evaluated in an emergency room or intensive care unit. Future studies should focus on confirming these associations in a prospective manner accounting for disease severity.

## Ethics Statement

This study was exempt from ethics committee approval because it presents a retrospective analysis of electrolyte data collected as part of clinician-driven care provided to patients at the institution hospital. No client or patient identifying information is presented.

## Author Contributions

RG conceived the study, analyzed data, and wrote the manuscript; SR and DF collected and analyzed data and edited the manuscript.

## Conflict of Interest Statement

The authors declare that the research was conducted in the absence of any commercial or financial relationships that could be construed as a potential conflict of interest.
