# Supplementary material for: Electrolyte Disturbances Are Associated with Non-Survival in Dogs—A Multivariable Analysis
Source: Front Vet Sci. 2017 Aug 18;4:135. doi: 10.3389/fvets.2017.00135 (PMC5563317; doi:10.3389/fvets.2017.00135)
Supplement: Supplementary file 1 [file Table_4.DOCX]

**Table 4. Summary case fatality and case number data for the whole population (n=33,117)**

| **[Na+]** | **Case fatality %** | **n** | **[K+]** | **Case fatality %** | **n** | **[Corr Cl-]** | **Case fatality %** | **n** | **[Ca2+]** | **Case fatality %** | **n** |
| --- | --- | --- | --- | --- | --- | --- | --- | --- | --- | --- | --- |
| 100-102 | 0.00 | 0 | 0.9-1.18 | - | 0 | 70-72 | - | 0 | 0.20-0.25 | 0.00 | 1 |
| 102-104 | 0.00 | 1 | 1.18-1.47 | 100.00 | 1 | 72-74 | 0 | 2 | 0.25-0.30 | 0.00 | 1 |
| 104-106 | 0.00 | 3 | 1.47-1.75 | 60.00 | 5 | 74-76 | 0 | 1 | 0.30-0.35 | 0.00 | 2 |
| 106-108 | - | 4 | 1.75-2.04 | 42.86 | 7 | 76-78 | - | 0 | 0.35-0.40 | 66.67 | 3 |
| 108-110 | 50.00 | 0 | 2.04-2.32 | 40.00 | 10 | 78-80 | 0 | 1 | 0.40-0.45 | 66.67 | 3 |
| 110-112 | 50.00 | 4 | 2.32-2.60 | 42.31 | 52 | 80-82 | 25 | 4 | 0.45-0.50 | 50.00 | 4 |
| 112-114 | 0.00 | 2 | 2.60-2.89 | 35.92 | 103 | 82-84 | 50 | 4 | 0.50-0.55 | 33.33 | 3 |
| 114-116 | 30.00 | 4 | 2.89-3.17 | 31.91 | 351 | 84-86 | 83.33 | 6 | 0.55-0.60 | 50.00 | 10 |
| 116-118 | 8.33 | 10 | 3.17-3.46 | 29.66 | 954 | 86-88 | 62.5 | 8 | 0.60-0.65 | 23.53 | 17 |
| 118-120 | 33.33 | 12 | 3.46-3.74 | 23.27 | 2321 | 88-90 | 33.33 | 9 | 0.65-0.70 | 60.00 | 15 |
| 120-122 | 28.57 | 15 | 3.74-4.02 | 18.07 | 5026 | 90-92 | 40 | 15 | 0.70-0.75 | 39.39 | 33 |
| 122-124 | 34.48 | 14 | 4.02-4.31 | 14.98 | 7304 | 92-94 | 27.59 | 29 | 0.75-0.80 | 41.86 | 43 |
| 124-126 | 31.71 | 29 | 4.31-4.59 | 15.22 | 6996 | 94-96 | 35.14 | 37 | 0.80-0.85 | 43.90 | 82 |
| 126-128 | 35.85 | 41 | 4.59-4.88 | 16.38 | 4861 | 96-98 | 31.82 | 66 | 0.85-0.90 | 39.80 | 98 |
| 128-130 | 35.71 | 53 | 4.88-5.16 | 19.43 | 2573 | 98-100 | 29.17 | 120 | 0.90-0.95 | 32.17 | 143 |
| 130-132 | 29.63 | 84 | 5.16-5.44 | 25.54 | 1151 | 100-102 | 26.78 | 183 | 0.95-1.00 | 32.86 | 213 |
| 132-134 | 28.64 | 135 | 5.44-5.73 | 29.07 | 571 | 102-104 | 26.76 | 355 | 1.00-1.05 | 35.05 | 331 |
| 134-136 | 31.98 | 206 | 5.73-6.01 | 29.07 | 293 | 104-106 | 20.55 | 871 | 1.05-1.10 | 36.38 | 503 |
| 136-138 | 30.04 | 369 | 6.01-6.30 | 35.84 | 164 | 106-108 | 17.65 | 1700 | 1.10-1.15 | 30.54 | 943 |
| 138-140 | 22.91 | 526 | 6.30-6.58 | 34.15 | 97 | 108-110 | 14.94 | 3099 | 1.15-1.20 | 27.02 | 1865 |
| 140-142 | 20.78 | 1004 | 6.58-6.86 | 32.99 | 74 | 110-112 | 14.87 | 4620 | 1.20-1.25 | 22.13 | 3810 |
| 142-144 | 19.44 | 1886 | 6.86-7.15 | 43.24 | 72 | 112-114 | 15.69 | 5373 | 1.25-1.30 | 19.82 | 6463 |
| 144-146 | 16.00 | 3261 | 7.15-7.43 | 43.06 | 34 | 114-116 | 16.15 | 5071 | 1.30-1.35 | 15.79 | 7994 |
| 146-148 | 14.69 | 5081 | 7.43-7.72 | 35.29 | 25 | 116-118 | 18.01 | 4220 | 1.35-1.40 | 13.10 | 6055 |
| 148-150 | 14.81 | 6108 | 7.72-8.00 | 56.00 | 17 | 118-120 | 22.43 | 3041 | 1.40-1.45 | 12.25 | 2703 |
| 150-152 | 16.65 | 5516 | 8.00-8.28 | 47.06 | 11 | 120-122 | 24.64 | 2041 | 1.45-1.50 | 13.43 | 901 |
| 152-154 | 18.96 | 3754 | 8.28-8.57 | 45.45 | 6 | 122-124 | 27.92 | 1182 | 1.50-1.55 | 12.28 | 334 |
| 154-156 | 25.98 | 2210 | 8.57-8.85 | 50.00 | 8 | 124-126 | 31.11 | 585 | 1.55-1.60 | 16.10 | 118 |
| 156-158 | 27.97 | 1170 | 8.85-9.14 | 12.50 | 6 | 126-128 | 37.01 | 254 | 1.60-1.65 | 25.30 | 83 |
| 158-160 | 30.00 | 622 | 9.14-9.42 | 0.00 | 3 | 128-130 | 25.56 | 90 | 1.65-1.70 | 19.23 | 52 |
| 160-162 | 34.25 | 350 | 9.42-9.70 | 0.00 | 6 | 130-132 | 15.79 | 38 | 1.70-1.75 | 20.59 | 34 |
| 162-164 | 34.19 | 219 | 9.70-9.99 | 33.33 | 3 | 132-134 | 28 | 25 | 1.75-1.80 | 30.30 | 33 |
| 164-166 | 47.83 | 155 | 9.99-10.27 | 33.33 | 3 | 134-136 | 13.33 | 15 | 1.80-1.85 | 13.33 | 30 |
| 166-168 | 46.88 | 92 | 10.27-10.56 | 0 | 1 | 136-138 | 33.33 | 15 | 1.85-1.90 | 24.14 | 29 |
| 168-170 | 55.00 | 64 | 10.56-10.84 | 100 | 1 | 138-140 | 11.11 | 18 | 1.90-1.95 | 19.23 | 26 |
| 170-172 | 51.61 | 40 | 10.84-11.12 | 0 | 1 | 140-142 | 20 | 15 | 1.95-2.00 | 25.93 | 27 |
| 172-174 | 50.00 | 31 | 11.12-11.41 | - | 0 | 142-144 | 0 | 4 | 2.00-2.05 | 33.33 | 21 |
| 174-176 | 40.00 | 16 | 11.41-11.69 | 100 | 1 |  |  |  | 2.05-2.10 | 30.00 | 20 |
| 176-178 | 0.00 | 10 | 11.69-11.98 | 0 | 1 |  |  |  | 2.10-2.15 | 22.22 | 18 |
| 178-180 | - | 3 | 11.98-12.26 | 0 | 1 |  |  |  | 2.15-2.20 | 11.11 | 9 |
| 180-182 | 50.00 | 2 | 12.26-12.54 | - | 0 |  |  |  | 2.20-2.25 | 0.00 | 8 |
| 182-184 | 75.00 | 4 | 12.54-12.83 | - | 0 |  |  |  | 2.25-2.30 | 62.50 | 8 |
| 184-186 | 0.00 | 2 | 12.83-13.11 | - | 0 |  |  |  | 2.30-2.35 | 30.00 | 10 |
| 186-188 | 100.00 | 2 | 13.11-13.40 | 0 | 1 |  |  |  | 2.35-2.40 | 40.00 | 10 |
| 188-190 | - | 0 | 13.40-13.68 | - | 0 |  |  |  | 2.40-2.45 | 50.00 | 4 |
| 190-192 | 0.00 | 1 | 13.68-13.96 | 0 | 1 |  |  |  | 2.45-2.50 | 0.00 | 1 |
| 192-194 | 100.00 | 1 | 13.96-14.25 | - | 0 |  |  |  | 2.50-2.55 | - | 0 |
| 194-196 | - | 0 | 14.25-14.53 | - | 0 |  |  |  | 2.55-2.60 | 33.33 | 3 |
| 196-198 | - | 0 | 14.53-14.82 | - | 0 |  |  |  |  |  |  |
| >198 | 100.00 | 1 | >14.82 | 0 | 1 |  |  |  |  |  |  |
