# Supplementary material for: Electrolyte Disturbances Are Associated with Non-Survival in Dogs—A Multivariable Analysis
Source: Front Vet Sci. 2017 Aug 18;4:135. doi: 10.3389/fvets.2017.00135 (PMC5563317; doi:10.3389/fvets.2017.00135)
Supplement: Supplementary file 2 [file Table_5.DOCX]

| **[Na+]** | **Case fatality %** | **n** | **[K+]** | **Case fatality %** | **n** | **[Corr Cl-]** | **Case fatality %** | **n** | **[Ca2+]** | **Case fatality %** | **n** |
| --- | --- | --- | --- | --- | --- | --- | --- | --- | --- | --- | --- |
| 100-102 | - | 0 | 0.9-1.18 | - | 0 | 70-72 | - | 0 | 0.20-0.25 | 0 | 1 |
| 102-104 | 0.00 | 1 | 1.18-1.47 | - | 0 | 72-74 | 0.00 | 2 | 0.25-0.30 | 0 | 1 |
| 104-106 | 0.00 | 3 | 1.47-1.75 | 0.00 | 2 | 74-76 | 0.00 | 1 | 0.30-0.35 | 0 | 2 |
| 106-108 | 0.00 | 4 | 1.75-2.04 | 23.08 | 4 | 76-78 | - | 0 | 0.35-0.40 | 50 | 2 |
| 108-110 | - | 0 | 2.04-2.32 | 12.00 | 6 | 78-80 | 0.00 | 1 | 0.40-0.45 | 0 | 1 |
| 110-112 | 0.00 | 2 | 2.32-2.60 | 9.13 | 39 | 80-82 | 25.00 | 4 | 0.45-0.50 | 0 | 2 |
| 112-114 | 50.00 | 2 | 2.60-2.89 | 8.96 | 75 | 82-84 | 0.00 | 2 | 0.50-0.55 | 0 | 2 |
| 114-116 | 0.00 | 4 | 2.89-3.17 | 5.42 | 263 | 84-86 | 50.00 | 2 | 0.55-0.60 | 16.67 | 6 |
| 116-118 | 0.00 | 7 | 3.17-3.46 | 3.63 | 737 | 86-88 | 0.00 | 3 | 0.60-0.65 | 0 | 13 |
| 118-120 | 0.00 | 11 | 3.46-3.74 | 2.51 | 1883 | 88-90 | 0.00 | 6 | 0.65-0.70 | 0 | 6 |
| 120-122 | 0.00 | 10 | 3.74-4.02 | 2.32 | 4273 | 90-92 | 0.00 | 9 | 0.70-0.75 | 0 | 20 |
| 122-124 | 0.00 | 10 | 4.02-4.31 | 2.14 | 6370 | 92-94 | 0.00 | 21 | 0.75-0.80 | 10.71 | 28 |
| 124-126 | 9.52 | 21 | 4.31-4.59 | 2.31 | 6072 | 94-96 | 0.00 | 24 | 0.80-0.85 | 11.54 | 52 |
| 126-128 | 3.45 | 29 | 4.59-4.88 | 4.14 | 4154 | 96-98 | 11.76 | 51 | 0.85-0.90 | 11.94 | 67 |
| 128-130 | 5.56 | 36 | 4.88-5.16 | 5.15 | 2122 | 98-100 | 6.59 | 91 | 0.90-0.95 | 11.01 | 109 |
| 130-132 | 10.00 | 60 | 5.16-5.44 | 6.93 | 894 | 100-102 | 6.29 | 143 | 0.95-1.00 | 11.73 | 162 |
| 132-134 | 8.65 | 104 | 5.44-5.73 | 10.00 | 427 | 102-104 | 5.11 | 274 | 1.00-1.05 | 8.12 | 234 |
| 134-136 | 5.77 | 156 | 5.73-6.01 | 8.45 | 202 | 104-106 | 3.22 | 715 | 1.05-1.10 | 10.36 | 357 |
| 136-138 | 3.46 | 260 | 6.01-6.30 | 10.64 | 120 | 106-108 | 2.44 | 1435 | 1.10-1.15 | 8.01 | 712 |
| 138-140 | 6.12 | 392 | 6.30-6.58 | 18.00 | 71 | 108-110 | 1.97 | 2689 | 1.15-1.20 | 6.59 | 1457 |
| 140-142 | 3.13 | 799 | 6.58-6.86 | 8.33 | 47 | 110-112 | 1.94 | 4011 | 1.20-1.25 | 4.9 | 3120 |
| 142-144 | 3.43 | 1547 | 6.86-7.15 | 21.43 | 50 | 112-114 | 2.18 | 4631 | 1.25-1.30 | 3.79 | 5386 |
| 144-146 | 3.63 | 2726 | 7.15-7.43 | 0.00 | 24 | 114-116 | 2.70 | 4370 | 1.30-1.35 | 2.19 | 6883 |
| 146-148 | 3.00 | 4400 | 7.43-7.72 | 14.29 | 14 | 116-118 | 3.57 | 3588 | 1.35-1.40 | 1.52 | 5343 |
| 148-150 | 2.51 | 5345 | 7.72-8.00 | 40.00 | 9 | 118-120 | 3.87 | 2454 | 1.40-1.45 | 1.82 | 2416 |
| 150-152 | 4.05 | 4824 | 8.00-8.28 | 0.00 | 7 | 120-122 | 6.79 | 1650 | 1.45-1.50 | 1.64 | 793 |
| 152-154 | 3.07 | 3228 | 8.28-8.57 | 0.00 | 5 | 122-124 | 8.39 | 930 | 1.50-1.55 | 1.68 | 298 |
| 154-156 | 3.81 | 1862 | 8.57-8.85 | 0.00 | 7 | 124-126 | 7.57 | 436 | 1.55-1.60 | 1.98 | 101 |
| 156-158 | 3.99 | 902 | 8.85-9.14 | 20.00 | 6 | 126-128 | 12.57 | 183 | 1.60-1.65 | 3.13 | 64 |
| 158-160 | 4.48 | 469 | 9.14-9.42 | 33.33 | 3 | 128-130 | 6.94 | 72 | 1.65-1.70 | 2.33 | 43 |
| 160-162 | 9.93 | 268 | 9.42-9.70 | 0.00 | 5 | 130-132 | 3.03 | 33 | 1.70-1.75 | 0 | 27 |
| 162-164 | 9.43 | 159 | 9.70-9.99 | - | 0 | 132-134 | 0.00 | 18 | 1.75-1.80 | 0 | 23 |
| 164-166 | 6.42 | 109 | 9.99-10.27 | 0.00 | 3 | 134-136 | 0.00 | 13 | 1.80-1.85 | 0 | 26 |
| 166-168 | 11.11 | 54 | 10.27-10.56 | 100.00 | 3 | 136-138 | 0.00 | 10 | 1.85-1.90 | 0 | 22 |
| 168-170 | 5.56 | 36 | 10.56-10.84 | - | 0 | 138-140 | 0.00 | 16 | 1.90-1.95 | 0 | 21 |
| 170-172 | 10.00 | 20 | 10.84-11.12 | - | 0 | 140-142 | 0.00 | 12 | 1.95-2.00 | 4.76 | 21 |
| 172-174 | 16.67 | 18 | 11.12-11.41 | 0.00 | 2 | 142-144 | 0.00 | 4 | 2.00-2.05 | 0 | 14 |
| 174-176 | 11.11 | 9 | 11.41-11.69 | 0.00 | 2 |  |  |  | 2.05-2.10 | 12.5 | 16 |
| 176-178 | 25.00 | 8 | 11.69-11.98 | - | 0 |  |  |  | 2.10-2.15 | 6.67 | 15 |
| 178-180 | 0.00 | 3 | 11.98-12.26 | - | 0 |  |  |  | 2.15-2.20 | 0 | 8 |
| 180-182 | - | 0 | 12.26-12.54 | - | 0 |  |  |  | 2.20-2.25 | 0 | 8 |
| 182-184 | 0.00 | 1 | 12.54-12.83 | 0.00 | 1 |  |  |  | 2.25-2.30 | 0 | 3 |
| 184-186 | 0.00 | 1 | 12.83-13.11 | - | 0 |  |  |  | 2.30-2.35 | 0 | 7 |
| 186-188 | 0.00 | 2 | 13.11-13.40 | 0.00 | 1 |  |  |  | 2.35-2.40 | 0 | 6 |
| 188-190 | 100.00 | 1 | 13.40-13.68 | - | 0 |  |  |  | 2.40-2.45 | 33.33 | 3 |
| 190-192 | - | 0 | 13.68-13.96 | - | 0 |  |  |  | 2.45-2.50 | - | 0 |
| 192-194 | 0.00 | 1 | 13.96-14.25 | - | 0 |  |  |  | 2.50-2.55 | 0 | 1 |
| 194-196 | - | 0 | 14.25-14.53 | 0.00 | 1 |  |  |  | 2.55-2.60 | 0 | 2 |
| 196-198 | - | 0 | 14.53-14.82 | - | 0 |  |  |  |  |  |  |
| >198 | - | 0 | >14.82 | - | 0 |  |  |  |  |  |  |

**Table 5. Summary case fatality and case number data after dogs that were euthanized were excluded (n=27,904)**
